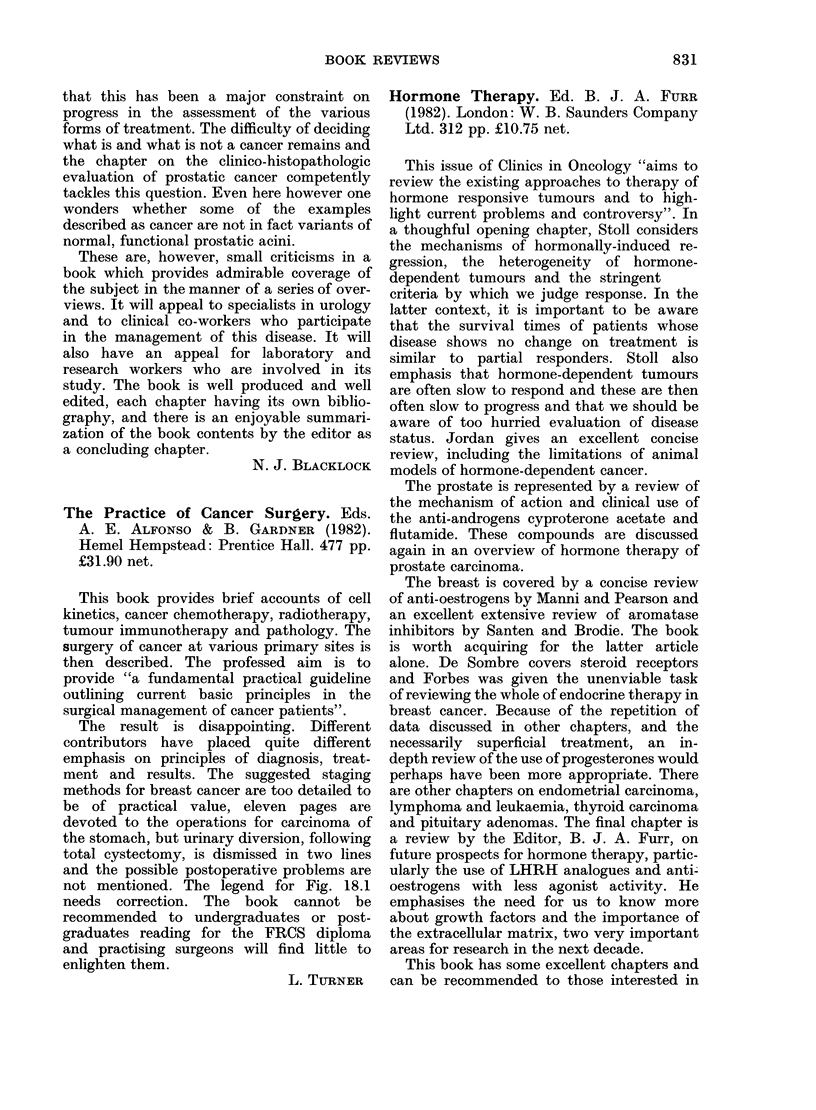# The Practice of Cancer Surgery

**Published:** 1982-11

**Authors:** L. Turner


**The Practice of Cancer Surgery.** Eds. A. E. ALFONSO & B. GARDNER (1982). Hemel Hempstead: Prentice Hall. 477 pp. £31.90 net.

This book provides brief accounts of cell kinetics, cancer chemotherapy, radiotherapy, tumour immunotherapy and pathology. The surgery of cancer at various primary sites is then described. The professed aim is to provide "a fundamental practical guideline outlining current basic principles in the surgical management of cancer patients".

The result is disappointing. Different contributors have placed quite different emphasis on principles of diagnosis, treatment and results. The suggested staging methods for breast cancer are too detailed to be of practical value, eleven pages are devoted to the operations for carcinoma of the stomach, but urinary diversion, following total cystectomy, is dismissed in two lines and the possible postoperative problems are not mentioned. The legend for Fig. 18.1 needs correction. The book cannot be recommended to undergraduates or postgraduates reading for the FRCS diploma and practising surgeons will find little to enlighten them.

L. TURNER